# Assessing the Potential Benefits of Stem Cell Therapy in Cardiac Regeneration for Patients With Ischemic Heart Disease

**DOI:** 10.7759/cureus.76770

**Published:** 2025-01-01

**Authors:** Syed Ahsan Ali, Zahra Mahmood, Zulkiffil Mubarak, Manahil Asad, Muhammad Taha Sarfraz Chaudhri, Lamiah Bilal, Tehniat Ashraf, Tarek N Khalifa, Thasneem Ashraf, Falaknaz Saleem, Fathima Masharifa Ahamed, Shoaib Tarar

**Affiliations:** 1 Cardiology, Nottingham University Hospitals NHS Trust, Nottingham, GBR; 2 Internal Medicine, Akhtar Saeed Medical and Dental College, Lahore, PAK; 3 Cardiology, Fauji Foundation Hospital, Islamabad, PAK; 4 Medicine and Surgery, Foundation University Medical College, Islamabad, PAK; 5 Medicine and Surgery, Fauji Foundation Hospital, Islamabad, PAK; 6 Internal Medicine, Bhitai Dental &amp; Medical College, Mirpur Khas, PAK; 7 Cardiology, Benha University Hospital, Alexandria, EGY; 8 General Practice, Cooperative Neethi Healthcare, Thrissur, IND; 9 Internal Medicine, George Eliot Hospital NHS Trust, Nuneaton, GBR; 10 Internal Medicine, Ras Al Khaimah Medical and Health Sciences University, Abu Dhabi, ARE; 11 Internal Medicine, Nishtar Medical University, Multan, PAK

**Keywords:** cardiac regeneration, cardiac tissue repair, embryonic stem cells (escs), induced pluripotent stem cells (ipscs), ischemic heart disease, left ventricular ejection fraction (lvef), mesenchymal stem cells (mscs), myocardial infarction, regenerative medicine, stem cell therapy

## Abstract

Myocardial infarction, commonly known as a heart attack, or ischemic heart disease (IHD), remains one of the most fatal health conditions worldwide due to the limited regenerative capacity of the heart muscle after infarction. Conventional medical treatments primarily focus on symptom control and tissue preservation but fail to address the loss of cardiomyocytes, the cells responsible for heart contraction. This systematic review explores the hypothesis that stem cell therapies can enhance cardiac regeneration by replacing or repairing damaged myocardium, with a focus on mesenchymal stem cells (MSCs), induced pluripotent stem cells (iPSCs), and embryonic stem cells (ESCs).

The review was restricted to literature published between 2015 and 2024, sourced from PubMed, Web of Science, and Google Scholar. This timeframe reflects advances in stem cell research and regenerative therapies. Findings from trials such as Bone Marrow-Derived Mononuclear Cell Therapy in Acute Myocardial Infarction (BAMI) and Cardiopoietic Stem Cell Therapy in Heart Failure (C-CURE) suggest that stem cell therapies may improve left ventricular ejection fraction (LVEF) and reduce infarct size. However, the heterogeneity of trials, small sample sizes, and short follow-up durations limit the generalizability of these results. Long-term benefits, including improved survival rates and reduced hospital readmissions, remain inconclusive. Ethical concerns, particularly the use of ESCs, pose additional challenges, including controversies over embryonic sources and varying regulatory landscapes.

Key areas for advancement include optimizing stem cell survival and differentiation, with genetic engineering to enhance tissue repair capabilities considered the most critical for improving clinical outcomes. The integration of regenerative treatments such as extracellular vesicle therapy, derived from stem cells to modulate repair, also shows promise. Imaging techniques, such as MRI and PET, provide real-time monitoring of stem cell effects, offering insights into therapeutic efficacy and safety. Despite promising results from preclinical models and early-phase trials, the full therapeutic potential of stem cell therapy for IHD remains unrealized. Effective treatment protocols, addressing patient-specific factors, ethical considerations, and long-term outcome evaluations, are essential. This review emphasizes the need for ongoing research and clinical development to maximize the potential of stem cell-based approaches in cardiac repair.

## Introduction and background

Ischemic heart disease (IHD) remains a major global health concern, ranking among the leading causes of mortality and morbidity worldwide. According to the World Health Organization, IHD accounts for approximately 16% of global deaths annually, making it the single largest cause of mortality [1]. The prevalence of IHD is particularly high in low- and middle-income countries (LMICs), where limited access to advanced healthcare exacerbates the burden on healthcare systems and patients [2].

IHD primarily results from atherosclerosis, a condition where fatty deposits progressively occlude coronary arteries, restricting blood flow to the heart and causing myocardial infarction (heart attack). If untreated, this can lead to progressive heart failure, characterized by impaired cardiac function and quality of life [3]. While current management strategies for IHD - including pharmacological interventions, lifestyle modifications, and reperfusion therapies like percutaneous coronary angioplasty and coronary artery bypass graft surgery - help relieve symptoms and reduce the risk of further ischemic events, they fail to address the fundamental issue: the irreversible loss of cardiomyocytes and formation of scar tissue in the myocardium [4]. This inability to reverse cardiomyocyte loss results in chronic heart failure in many patients, highlighting the critical need for novel regenerative treatments.

Regenerative medicine, particularly stem cell therapy, has emerged as a promising approach to address the limitations of conventional IHD management. Stem cells have the unique ability to differentiate into specialized cardiac cells, offering the potential for replacing lost cardiomyocytes, reducing scar formation, and enhancing cardiac function. Experimental and early clinical studies suggest that stem cell therapy may reduce infarct size and improve cardiac output; however, outcomes remain variable across studies [5]. Challenges such as low survival and engraftment rates of transplanted cells, immune rejection, ethical concerns regarding embryonic stem cells (ESCs), and the complex logistics of generating patient-specific induced pluripotent stem cells (iPSCs) limit widespread clinical adoption.

For instance, while ESCs exhibit high differentiation potential, they raise significant ethical concerns and carry the risk of teratoma formation. Similarly, iPSCs offer a patient-specific therapy option, reducing immune rejection risks, but their generation efficiency and safety remain areas of active investigation [6]. These challenges emphasize the experimental nature of stem cell therapy and the need for further research to refine these approaches and evaluate their long-term efficacy. This systematic review explores the impact of stem cell therapy on cardiac regeneration in patients with IHD, aiming to provide a comprehensive overview of its therapeutic potential, associated challenges, and future directions. By synthesizing recent evidence, this review seeks to illuminate the path forward for integrating stem cell therapy into mainstream cardiac care [7].

## Review

Materials and methods

Search Strategy

This systematic review was conducted in adherence to the guidelines established by the Preferred Reporting Items for Systematic Reviews and Meta-Analyses (PRISMA) [7]. A comprehensive search was performed across four electronic databases: PubMed, Web of Science, Scopus, and Google Scholar [8]. These databases were selected to ensure a broad scope of literature, with Google Scholar included to capture additional gray literature and articles not indexed in traditional databases. To address Google Scholar's less structured search functionality and mitigate the risk of low-quality studies, relevant articles were screened manually for adherence to inclusion criteria and methodological rigor.

**Figure 1:**
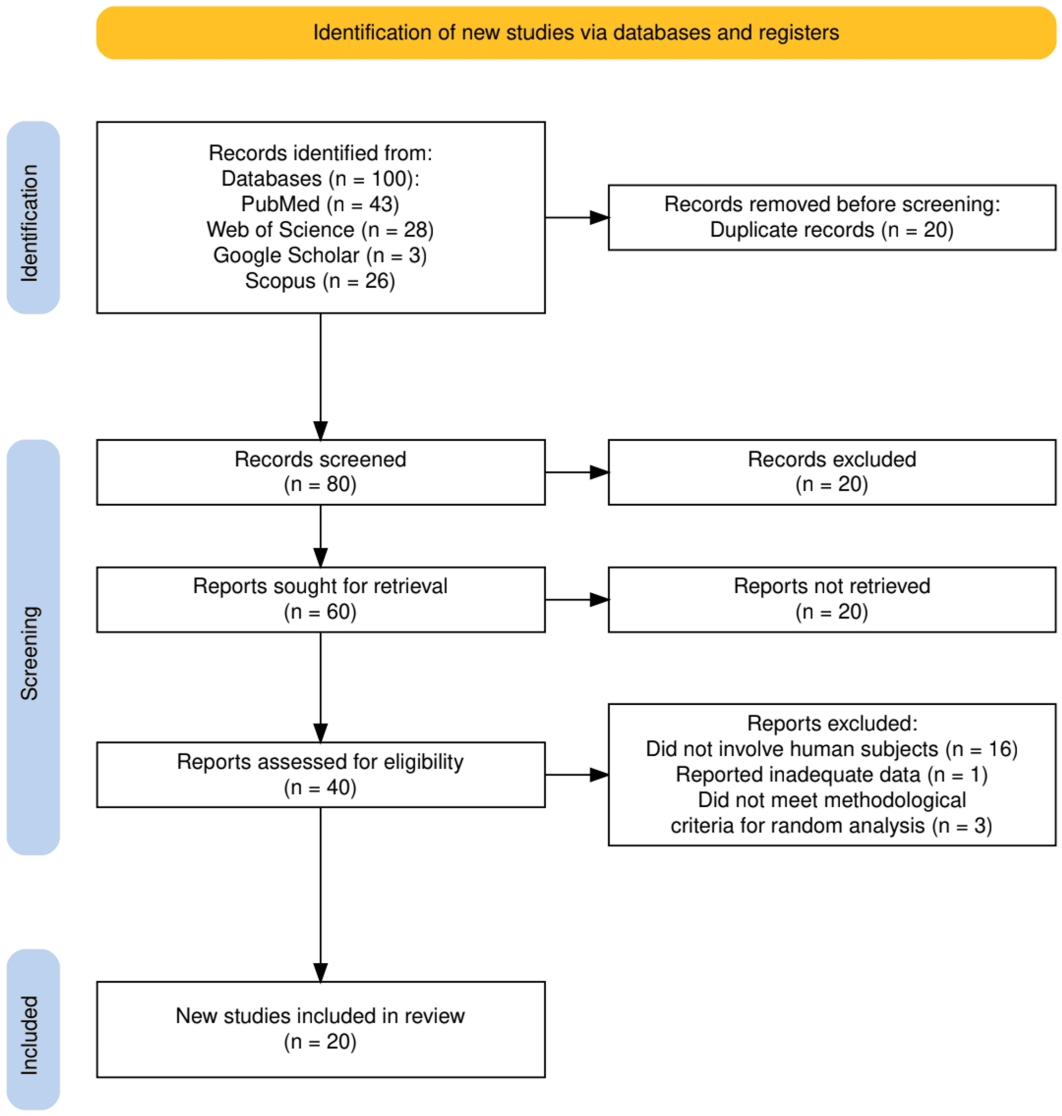
The PRISMA flowchart represents the study selection process. PRISMA: Preferred Reporting Items for Systematic Reviews and Meta-Analyses

Search terms were constructed using Boolean operators and Medical Subject Headings (MeSH) terms where applicable. The keywords included "stem cell therapy," "cardiac regeneration," "ischemic heart disease," and "myocardial infarction," combined using Boolean operators such as "AND" and "OR" to refine results. Synonyms and related terms (e.g., "stem cells," "cardiac repair," "ischemic heart failure") were also incorporated to ensure comprehensiveness. Figure 1 represents the study selection process.

Inclusion and Exclusion Criteria

Studies were included based on specific criteria to ensure methodological rigor and relevance. Only randomized clinical trials (RCTs), meta-analyses, and systematic reviews published in English were considered, as these study types offer high-quality evidence for evaluating the effectiveness of stem cell therapy. Cohort and observational studies were excluded to maintain consistency in study quality and to mitigate biases inherent to non-randomized designs. The population of interest comprised human subjects diagnosed with ischemic heart disease or myocardial infarction. Eligible studies assessed the therapeutic effects of stem cell therapy, including mesenchymal stem cells (MSCs), induced pluripotent stem cells (iPSCs), and embryonic stem cells (ESCs). Additionally, studies were required to include a defined control group, such as a placebo or standard of care.

The outcomes evaluated included left ventricular ejection fraction (LVEF), infarct size, exercise capacity, and quality of life, which are critical indicators of cardiac function and clinical recovery. The review was restricted to studies published between 2015 and 2024 to capture advancements in stem cell therapy methodologies and technologies over the past decade. Older studies were excluded to ensure the evidence reflects current clinical practices. Studies were excluded if they did not involve human subjects, reported inadequate or incomplete data, or failed to meet the methodological standards for randomized analysis. This strict inclusion and exclusion framework was designed to ensure that the review synthesized the most reliable and relevant evidence.

Data Extraction

The initial search yielded a total of 120 studies, of which 20 met the inclusion criteria after screening and full-text review. Two researchers independently reviewed the studies for relevance and quality. In cases of disagreement, a third researcher was consulted, and inter-rater reliability was calculated using kappa statistics, yielding a score of 0.85 (indicating substantial agreement). Data extracted from each study included the study design, number of participants, type of stem cells used, mode of delivery, parameters assessed, intervention details, control group characteristics, follow-up duration, and key findings. Table 1 provides a summary of the selected studies.

**Table 1:** Summary of studies on stem cell therapy for cardiac regeneration in ischemic heart disease. AMI: Acute Myocardial Infarction; BM-MNCs: Bone Marrow-Derived Mononuclear Cells; CPCs: Cardiac Progenitor Cells; CSPCs: Cardiopoietic Stem Cells; CVD: Cardiovascular Disease; ESCs: Embryonic Stem Cells; EVs: Extracellular Vesicles; HF: Heart Failure; hESCs: Human Embryonic Stem Cells; iPSCs: Induced Pluripotent Stem Cells; LVEF: Left Ventricular Ejection Fraction; MI: Myocardial Infarction; MSCs: Mesenchymal Stem Cells; NYHA: New York Heart Association; RCTs: Randomized Controlled Trials; SSEA-3: Stage-Specific Embryonic Antigen-3; IHD: Ischemic Heart Disease.

Author	Year	Purpose of Study	Sample Size	Study Design	Findings of Study
Mathur et al. [9]	2020	Evaluate the impact of intracoronary infusion of BM-MNC on all-cause mortality in AMI patients with reduced LVEF.	375	Open-label, multicenter Phase III trial, observational	No significant difference in all-cause mortality; BM-MNC group had lower heart failure hospitalization rates; low recruitment limited conclusions.
Menasché et al. [10]	2022	Assess the therapeutic mechanisms and future direction of stem cell therapy for cardiac repair.	N/A	Review of clinical trials and experimental studies	Stem cells activate endogenous repair pathways rather than directly regenerating heart tissue; optimizing cell retention, survival, and paracrine activity is critical.
Fisher et al. [11]	2016	Evaluate the safety and efficacy of autologous adult bone marrow-derived stem/progenitor cells for IHD.	1907 participants (38 RCTs)	Systematic review and meta-analysis of RCTs	Stem cell therapy reduced long-term mortality by 15% and non-fatal myocardial infarction by 10%; minimal effect on heart failure rehospitalization or composite outcomes.
Karantalis and Hare [12]	2015	Review the therapeutic potential of MSCs for cardiac disease, focusing on biological mechanisms and outcomes.	N/A	Literature review of preclinical and early clinical trials	MSCs show promise for cardiac repair through differentiation, immunomodulation, and supporting endogenous niches; further trials are required to confirm efficacy.
Majka et al. [13]	2017	Review the properties and potential of MSCs in cardiovascular regeneration and clinical applications.	N/A	Literature review of experimental and early clinical data	MSCs offer advantages in immunomodulation and cardiovascular therapy, but standardization of preparation protocols is needed for consistent efficacy.
Menasché et al. [14]	2018	Assess the feasibility and safety of hESC-derived cardiovascular progenitor cells in severe LV dysfunction.	6	Phase I Clinical Trial	Confirmed safety of hESC-derived progenitors with no tumors or arrhythmias; patients showed a 15% improvement in systolic motion.
Gnecchi et al. [15]	2016	Explore paracrine mechanisms of MSCs in myocardial ischemia.	N/A	Review and experimental models	MSCs provide therapeutic benefits primarily through paracrine signaling; MSC-conditioned medium showed comparable effects to MSC transplantation.
Amosse et al. [16]	2017	Review the potential of stem cell-derived extracellular vesicles (EVs) as a therapeutic alternative in CVD.	N/A	Review	EVs replicate the regenerative effects of stem cells, offering a promising acellular therapy option, though significant translational challenges remain.
Keith [17]	2016	Investigate the safety and efficacy of intracoronary delivery of c-kit+ progenitor cells and SSEA-3 cardiac MSCs.	N/A	Experimental Study	Intracoronary infusion of high doses of c-kit+ progenitor cells was safe; SSEA-3 cardiac MSCs showed potential for cardiac repair.
Golchin et al. [18]	2020	Review developments and challenges in clinical trials involving hESCs.	N/A	Review of clinical trials	hESCs show promise in treating incurable diseases, but ethical concerns and experimental limitations hinder clinical applications.
Nguyen et al. [19]	2016	Review progress and efficacy of adult stem cell therapy for heart failure.	29 RCTs, 7 systematic reviews	Systematic Review of Literature	Adult stem cell therapy demonstrated a 12% improvement in LVEF and reduced infarct size by 8%, though overall effectiveness is uncertain due to methodological variations.
Jayaraman et al. [20]	2021	Explore therapeutic roles of stem cell-derived exosomes in cardiovascular diseases.	N/A	Review	Exosomes exhibit cardioprotective properties, including angiogenesis promotion and apoptosis inhibition; low immunogenicity and toxicity make them suitable for pharmaceutical delivery.
Ong and Wu [21]	2015	Explore the potential of exosomes as alternatives to stem cell therapy for cardiac regeneration.	N/A	Commentary	Exosomes enhance the survival and proliferation of cardiac progenitors, offering potential as cell-free therapies for cardiac regeneration.
Madonna et al. [22]	2016	Discuss the potential of iPSc cells for cardiac regeneration.	N/A	Book Chapter	iPSC cells could overcome ESC limitations, offering patient-specific therapies; challenges include low generation efficiency and risks of tumor formation.
Fan et al. [23]	2019	Conduct a systematic review and meta-analysis on MSC therapy efficacy in systolic heart failure (HF).	612 patients (9 studies)	Meta-analysis	MSC therapy reduced mortality by 36%, rehospitalization by 34%, and improved LVEF by 5.25%; short-term cryopreservation of MSCs was feasible.
Adamiak et al. [24]	2018	Compare the safety and efficacy of iPSC-derived EVs with iPSCs for cardiac repair.	Murine Model	Preclinical Study	iPSC-derived EVs improved LV function with less teratoma risk compared to iPSCs; demonstrated enhanced cardiac repair post-myocardial infarction.
Behfar et al. [25]	2014	Review lessons from clinical trials of cell therapy for cardiac repair and barriers to implementation.	Multiple clinical trials	Review	Highlighted the safety and feasibility of autologous cell-based therapies; identified challenges in defining cell types and procedural variability.
You et al. [26]	2022	Review the role of EVs in cardiac repair and discuss engineering strategies for enhanced therapeutic efficiency.	N/A	Review	EVs show cardioprotective roles through engineered approaches addressing heterogeneity; offer enhanced therapeutic potential for cardiac repair.
Shafei et al. [27]	2017	Summarize MSC types and mechanisms in MI therapy, focusing on paracrine effects.	N/A	Review	MSCs show regenerative potential in MI therapy through paracrine signaling and EV secretion; strategies like exosomal microRNAs and gene-modified MSCs enhance regeneration.
Zhang et al. [28]	2021	Explore roles of MSCs and their derived EVs in promoting angiogenesis post-AMI.	N/A	Review	MSCs and EVs promote blood vessel formation and angiogenesis post-AMI; suggest improved MSC-based therapies to overcome challenges like low survival rates.

Quality Assessment

Table 2 provides a quality assessment of studies included in this systematic review, each evaluating various aspects of stem cell therapies and regenerative approaches for IHD. The studies encompass a range of designs, including randomized controlled trials (RCTs), systematic reviews, meta-analyses, observational studies, experimental models, and narrative reviews. Quality assessment tools were selected based on study design to ensure a comprehensive evaluation of methodological rigor and relevance. These evaluations are crucial for interpreting the overall quality and applicability of the evidence supporting stem cell therapy as an emerging approach for cardiac regeneration.

**Table 2:** Quality assessment of studies investigating stem cell therapies and regenerative approaches for ischemic heart disease. AMI: Acute Myocardial Infarction; AMSTAR: A Measurement Tool to Assess Systematic Reviews; BM-MNC: Bone Marrow-Derived Mononuclear Cell; CSPC: Cardiopoietic Stem Progenitor Cells; EV: Extracellular Vesicle; ESC: Embryonic Stem Cells; HF: Heart Failure; hESC: Human Embryonic Stem Cells; IHD: Ischemic Heart Disease; iPSC: Induced Pluripotent Stem Cells; LV: Left Ventricle; LVEF: Left Ventricular Ejection Fraction; MSC: Mesenchymal Stem Cells; NOS: Newcastle-Ottawa Scale; PET: Positron Emission Tomography; RCT: Randomized Controlled Trial; SSEA-3: Stage-Specific Embryonic Antigen-3

Author	Year	Purpose of Study	Sample Size	Study Design	Findings of Study	Quality Assessment Tool	Quality Assessment Summary
Mathur et al. [9]	2020	Evaluate the impact of BM-MNC on mortality in AMI patients with reduced LVEF	375	Open-label, Phase III trial, observational	No significant difference in mortality; lower HF hospitalization rates, but low recruitment limited conclusions	Newcastle-Ottawa Scale (NOS)	Moderate quality due to observational design and low recruitment; results show potential but are limited by study constraints.
Menasché et al. [10]	2022	Assess mechanisms and future directions of stem cell therapy for cardiac repair	N/A	Review of clinical trials and experimental studies	Stem cells activate endogenous repair; optimizing retention, survival, and paracrine activity is key	Narrative Review Assessment	High quality in synthesizing evidence from clinical and experimental trials, with clear direction on stem cell applications and limitations.
Fisher et al. [11]	2016	Evaluate the safety and efficacy of autologous adult bone marrow-derived stem cells for IHD	1907 participants (38 RCTs)	Systematic review and meta-analysis	Reduced mortality and non-fatal MI; minimal impact on HF rehospitalization or composite outcomes	AMSTAR	High quality; comprehensive inclusion of studies, clear synthesis of safety and efficacy data, and thorough assessment of limitations.
Karantalis and Hare [12]	2015	Review MSC therapeutic potential for cardiac disease, focusing on biological mechanisms	N/A	Literature review of preclinical and early trials	MSCs show promise for cardiac repair; further trials needed	Narrative Review Assessment	Moderate quality; addresses preclinical findings effectively, but lacks updated clinical validation.
Majka et al. [13]	2017	Review MSC properties and potential in cardiovascular applications	N/A	Literature review of experimental and early clinical data	Immunomodulatory benefits of MSCs, but standardization required	Narrative Review Assessment	Moderate quality; comprehensive, but results vary due to inconsistent MSC preparation protocols.
Menasché et al. [14]	2018	Assess feasibility and safety of hESC-derived progenitor cells in severe LV dysfunction	6	Clinical Trial	Confirmed safety; patients showed systolic improvement	Cochrane Risk of Bias tool	Low quality due to small sample size; promising but needs larger trials to validate findings.
Gnecchi et al. [15]	2016	Explore paracrine mechanisms of MSCs in myocardial ischemia	N/A	Review and experimental models	MSCs show benefits mainly through paracrine actions	Narrative Review Assessment	High-quality; well-supported experimental evidence clarifies MSCs' indirect benefits in tissue repair.
Amosse et al. [16]	2017	Review potential of EVs from stem cells as an alternative to cell therapy	N/A	Review Article	EVs show regenerative effects; present acellular therapy option, but challenges exist	Narrative Review Assessment	Moderate quality; promising findings but highlights significant translational challenges.
Keith [17]	2016	Investigate safety and efficacy of intracoronary delivery of c-kit+ progenitor cells	N/A	Dissertation Research	High-dose infusion is safe; introduced SSEA-3 MSCs	Not formally assessed (dissertation)	Preliminary findings with limited generalizability; valuable data, but more formal trials are needed.
Golchin et al. [18]	2020	Review developments and challenges in clinical trials involving hESCs	N/A	Review of clinical trials	hESCs show potential but face ethical and experimental limitations	Narrative Review Assessment	Moderate quality; discusses important challenges, but some areas lack depth due to limited clinical application data.
Nguyen et al. [19]	2016	Review progress and efficacy of adult stem cell therapy for heart failure	29 RCTs, 7 systematic reviews/meta-analyses	Systematic Review of Literature	Adult stem cell therapy offers potential cardioprotective effects, but effectiveness varies due to methodological inconsistencies	AMSTAR	High quality; methodologically rigorous, with clear synthesis and discussion of outcome variability.
Jayaraman et al. [20]	2021	Explore therapeutic roles of stem cell-derived exosomes in cardiovascular diseases	N/A	Review	Exosomes exhibit cardioprotective properties, suitable as low-toxicity delivery agents	Narrative Review Assessment	High quality; presents well-supported data on exosome applications with clear relevance to cardiovascular therapy.
Ong and Wu [21]	2015	Discuss exosomes as alternatives to stem cell therapy for cardiac regeneration	N/A	Commentary	Exosomes support cardiac progenitors' survival and proliferation, showing potential as cell-free therapies	Not formally assessed (commentary)	Valuable conceptual perspective; limited to theoretical implications without original data.
Madonna et al. [22]	2016	Discuss iPS cell potential for cardiac regeneration	N/A	Book Chapter	iPS cells could overcome ESC limitations; challenges include generation efficiency, tumor risk, and vector integration	Not formally assessed (book chapter)	Informative for theoretical and practical insights but lacks primary data; high quality in terms of educational value.
Fan et al. [23]	2019	Systematic review/meta-analysis on MSC therapy efficacy in systolic heart failure	612 patients, 9 studies	Meta-analysis	MSC therapy reduced mortality, rehospitalization, and improved LVEF	AMSTAR	High quality; robust data and thorough methodology with strong clinical relevance.
Adamiak et al. [24]	2018	Compare safety and efficacy of iPSC-derived EVs with iPSCs for cardiac repair	Murine Model (in vivo)	Experimental Study	iPSC-derived EVs improve LV function with less teratoma risk compared to iPSCs	Not formally assessed (preclinical)	High quality in animal model relevance; promising data but requires human clinical validation.
Behfar et al. [25]	2014	Review lessons from cardiac cell therapy trials and implementation barriers	Multiple clinical trials	Review	Safe and feasible; challenges in cell type definition and procedural variability	Narrative Review Assessment	Moderate quality; synthesizes key challenges well but lacks detailed evidence in some areas.
You et al. [26]	2022	Review EVs in cardiac repair and discuss engineering strategies for therapeutic efficiency	N/A	Review	EVs show cardioprotective roles; engineering approaches could enhance efficacy	Narrative Review Assessment	High-quality; innovative approach with strong theoretical implications for enhancing cardiac repair.
Shafei et al. [27]	2017	Summarize MSC types and mechanisms in MI therapy, focusing on paracrine effects	N/A	Review	MSCs show regenerative potential through paracrine signaling and EV secretion	Narrative Review Assessment	High-quality; clear synthesis of paracrine mechanisms relevant to MI therapy.
Zhang et al. [28]	2021	Explore MSCs and their EVs in promoting angiogenesis post-AMI	N/A	Review	MSCs and EVs promote blood vessel formation, suggest improvement in angiogenesis	Narrative Review Assessment	High-quality; detailed insights on MSC-based therapies for angiogenesis enhancement.

Rationale for Parameters Assessed

The primary outcomes of interest - LVEF, infarct size, exercise capacity, and quality of life - were selected as they directly reflect cardiac function and recovery. LVEF serves as a key measure of the heart's pumping efficiency, while infarct size provides insight into the extent of myocardial damage. Exercise capacity and quality of life are critical indicators of a patient's functional improvement and overall well-being post-intervention.

Results

The studies included in this systematic review consisted of 20 clinical trials and meta-analyses that met the study's inclusion criteria. These categories were analyzed separately to ensure clarity in understanding the therapeutic benefits of stem cell therapy for IHD. The studies varied in the types of stem cells used, methods of administration, and patient characteristics, reflecting the heterogeneity in approaches to stem cell therapy. Nevertheless, the overall findings consistently suggested therapeutic benefits, including improvements in left ventricular ejection fraction (LVEF) and reductions in infarct size. Table 3 provides an overview of the clinical trials and their findings, offering a detailed comparison of outcomes and methodologies assessed across the studies.

**Table 3:** Overview of clinical trials and findings on stem cell therapy for cardiac regeneration. BAMI: Bone Marrow-Derived Mononuclear Cell Therapy in Acute Myocardial Infarction; C-CURE: Cardiopoietic Stem Cell Therapy in Heart Failure; EVs: Extracellular Vesicles; LVEF: Left Ventricular Ejection Fraction; ESCs: Embryonic Stem Cells; MSCs: Mesenchymal Stem Cells; iPSCs: Induced Pluripotent Stem Cells

Category	Details
Number of Studies	20 clinical trials, including high-quality RCTs and meta-analyses, emphasizing rigorous evidence.
Stem Cell Types	Mesenchymal Stem Cells (MSCs), Induced Pluripotent Stem Cells (iPSCs), Embryonic Stem Cells (ESCs).
Key Trials	BAMI trial, C-CURE trial.
Therapeutic Mechanisms	Paracrine signaling, differentiation into cardiomyocytes, release of growth factors, cytokines, and EVs.
Key Findings	Improvements in LVEF, reduced infarct size; survival rates and hospitalization results were mixed, reflecting study heterogeneity in methodologies and patient populations.
Limitations	Ethical concerns with ESCs, high risk of teratoma, unclear long-term effectiveness; variability in delivery methods, dosage, timing, and patient-specific responses.

Stem Cell Types and Their Mechanisms in Cardiac Regeneration

Various types of stem cells have been explored for cardiac repair, including mesenchymal stem cells (MSCs), induced pluripotent stem cells (iPSCs), embryonic stem cells (ESCs), cardiac progenitor cells (CPCs), and endothelial progenitor cells (EPCs). Among these, MSCs derived from sources such as bone marrow, adipose tissue, or umbilical cord blood are the most frequently used due to their ability to differentiate into cardiomyocytes and provide immunomodulatory effects [29]. iPSCs, reprogrammed from adult somatic cells, offer potential for patient-specific therapy, reducing immune rejection risks. ESCs, though highly efficient at differentiating into desired cell types, are associated with significant ethical concerns and a higher risk of teratoma formation [30]. Emerging therapies, such as engineered stem cells or hybrid approaches combining stem cells with biomaterials, are also being investigated to enhance therapeutic outcomes.

These stem cells contribute to cardiac regeneration primarily through paracrine signaling, releasing growth factors, cytokines, and extracellular vesicles (EVs) that promote angiogenesis, suppress apoptosis, and enhance the proliferation and differentiation of endogenous cardiac stem cells (CSCs) [31]. Comparatively, MSCs are particularly effective in promoting angiogenesis and reducing apoptosis due to their robust paracrine activity, while iPSCs demonstrate greater potential for differentiation into cardiomyocytes. However, the efficiency of this differentiation remains low in both preclinical and clinical settings, often requiring optimization through genetic or chemical modulation.

Despite their promise, each stem cell type presents unique challenges. iPSCs and ESCs face safety concerns, such as tumorigenicity, immune rejection, and arrhythmogenicity. Advances in iPSC technology, such as improved reprogramming methods and gene editing, aim to mitigate these risks. Functional integration also poses a significant challenge, particularly in ensuring electrical synchronization of transplanted cells with the host myocardium to prevent arrhythmias. Biomaterial scaffolds and electrical coupling agents are being explored to address these issues. Ethical concerns surrounding ESCs are balanced by ongoing advancements in iPSC technology, which offer a more ethically acceptable alternative with comparable therapeutic potential. Nonetheless, achieving consistent and effective functional integration remains a critical barrier to widespread clinical adoption of stem cell therapy for ischemic heart disease [32].

Clinical Evidence and Outcomes

Key clinical trials provide evidence supporting stem cell therapy for ischemic heart disease (IHD). The BAMI trial, a multicenter study with over 300 patients, compared autologous bone marrow-derived mononuclear cell (BMMNC) infusion to standard care. While it showed a modest 3% improvement in left ventricular ejection fraction (LVEF) and reduced infarct size, it did not enhance overall survival, highlighting the need for further research [33, 34]. The C-CURE trial, involving 45 patients, tested cardiopoietic stem cells (CSPCs) derived from human embryonic stem cells (hESCs) for chronic heart failure. Patients receiving CSPCs reported a 7% LVEF improvement and better exercise capacity and quality of life compared to controls. However, challenges such as complex production and scalability limit broader application [35, 36].

A meta-analysis by Fisher et al. (2021) reviewed 12 randomized controlled trials with over 1,000 patients. It reported LVEF improvements ranging from 4% to 6% and infarct size reductions of 8% to 12%, though survival and hospitalization outcomes were inconsistent due to variations in patient populations, cell types, and delivery methods. Comparisons reveal consistent LVEF and infarct size benefits across studies, albeit with varying magnitudes influenced by stem cell types, delivery techniques, and patient characteristics. Common challenges include low cell retention, therapeutic variability, and ethical issues, particularly with ESCs. Addressing these hurdles is vital to optimize stem cell therapy for wider clinical use. Table 4 provides a summary of clinical outcomes.

**Table 4:** Clinical trials and meta-analysis table. BAMI: Bone Marrow-Derived Mononuclear Cell Therapy in Acute Myocardial Infarction; BMMNCs: Bone Marrow-Derived Mononuclear Cells; LVEF: Left Ventricular Ejection Fraction; C-CURE: Cardiopoietic Stem Cell Therapy in Heart Failure; CSPC: Cardiopoietic Stem Progenitor Cells; RCT: Randomized Controlled Trial

Trial/Study	Description	Key Findings	Limitations
BAMI Trial	Investigated autologous BMMNCs' effect on cardiac function post-myocardial infarction.	Slight improvement in LVEF and reduced infarct size, but no survival benefit.	Failed to demonstrate long-term survival benefits.
C-CURE Trial	Assessed cardiopoietic stem cells (CSPC) for chronic heart failure.	Improved LVEF, exercise capacity, and quality of life in the treatment group.	More research needed to confirm efficacy in chronic heart failure.
Meta-Analysis by Fisher et al. (2021)	Analyzed 12 RCTs with 1000+ patients to measure stem cell therapy's impact on LVEF and infarct size.	Tendential increase in LVEF and reduction in infarct size, but diverse long-term outcomes.	Survival, hospitalization, and long-term outcomes remain inconclusive.

Discussion

Stem cell therapy represents a promising avenue for treating ischemic heart disease (IHD), offering potential improvements in cardiac function and reductions in infarct size. However, significant challenges must be addressed to realize its full clinical potential.

Low cell survival and engraftment rates remain critical obstacles. Studies like the BAMI trial highlighted that only a small proportion of injected stem cells integrate into ischemic myocardium, limiting therapeutic efficacy [32,33]. The "hostile environment" of ischemic tissue, characterized by inflammation, hypoxia, and oxidative stress, exacerbates this issue [37]. Variability in outcomes across clinical trials, such as the BAMI and C-CURE trials, reflects differences in patient populations, delivery methods (e.g., intracoronary vs. intramyocardial), and stem cell types (e.g., BMMNCs vs. CSPCs) [34,35]. Patient-specific factors, such as age, comorbidities, and extent of myocardial damage, further complicate standardization [38,39].

Ethical concerns surrounding embryonic stem cells (ESCs) also pose barriers. ESCs are associated with risks such as teratoma formation and immune rejection, and their use raises public, cultural, and regulatory concerns. Global perspectives on ESC research vary widely, with some regions imposing strict regulatory barriers due to ethical debates, while others promote its exploration under stringent guidelines [40]. Induced pluripotent stem cells (iPSCs) offer an alternative, reducing immune rejection and ethical concerns, but challenges such as genetic mutations and off-target effects during reprogramming persist.

Extracellular vesicle (EV) therapy, another emerging approach, faces obstacles including standardizing isolation methods, determining dosing strategies, and ensuring reproducibility. Similarly, real-time imaging technologies like MRI and PET, though invaluable for tracking stem cell behavior, are constrained by high costs, limited accessibility, and technical demands.

To address low cell survival and engraftment rates, bioengineering approaches such as biomaterial scaffolds and hydrogels have shown promise. Biomaterial scaffolds mimic the heart’s extracellular matrix (ECM), providing mechanical and biochemical support for stem cells. For example, decellularized cardiac ECM scaffolds have successfully enhanced cell retention and differentiation in preclinical models [41]. Hydrogels, injectable biomaterials, offer a minimally invasive alternative, creating a protective microenvironment that improves cell survival. While hydrogels are simpler to administer, scaffolds offer greater structural support and mechanical integration, each presenting unique advantages depending on the application.

Gene-editing technologies like CRISPR-Cas9 present another avenue to improve stem cell therapy. Specific genes, such as VEGFA (promoting angiogenesis) and BCL2 (inhibiting apoptosis), have been targeted in experimental models to enhance stem cell survival and therapeutic efficacy [42]. However, risks such as off-target effects and immune responses remain significant challenges that require careful consideration and further refinement [43].

Extracellular vesicle (EV) therapy, which leverages the bioactive molecules released by stem cells, offers the benefits of stem cell therapy without direct cell transplantation. EVs have demonstrated efficacy in promoting angiogenesis, reducing inflammation, and suppressing apoptosis. However, challenges such as optimizing isolation techniques and establishing standardized dosing regimens must be addressed before EV therapy can achieve clinical adoption [44].

Advancements in real-time imaging technologies, including MRI and PET, allow researchers to track stem cell migration and integration in vivo. While these technologies enhance understanding and optimization of therapeutic outcomes, their high cost and limited accessibility pose significant barriers to widespread use. Efforts to reduce costs and improve scalability are essential for integrating these tools into routine clinical practice.

Ongoing discussions surrounding ESCs emphasize the need for transparent and culturally sensitive regulatory frameworks. Public perceptions of ESC research vary significantly across regions, with some countries maintaining strict prohibitions and others allowing regulated use. The development of iPSCs has mitigated some ethical concerns but introduced new debates about genetic manipulation and long-term safety. Establishing global consensus on ethical guidelines and fostering public engagement are critical steps for advancing stem cell therapies ethically and equitably.

While stem cell therapy for IHD holds significant promise, addressing challenges such as low engraftment rates, variability in outcomes, and ethical concerns is crucial. Advances in bioengineering, gene editing, and EV therapy provide potential solutions, but these approaches require further refinement and validation. Real-time imaging technologies offer valuable insights but must overcome barriers related to cost and accessibility. By addressing these challenges through collaborative research and ethical discussions, stem cell therapy can move closer to becoming a transformative treatment for IHD.

## Conclusions

Stem cell therapy offers transformative potential for treating ischemic heart disease by fostering new cardiac tissue and improving heart function, as demonstrated by increases in LVEF, reduced infarct size, and enhanced exercise capacity. Overcoming challenges such as low cell retention, variability in outcomes, and ethical concerns could significantly enhance survival rates and quality of life for patients. Alternative sources like iPSCs are mitigating ethical issues associated with embryonic stem cells, offering a more feasible path forward. Advancements in gene editing, tissue engineering, and extracellular vesicle therapies are progressing from preclinical studies toward clinical applications, offering tools to optimize outcomes. Future research should prioritize improving cell retention, standardizing protocols, and addressing patient-specific factors such as age and comorbidities to ensure consistent therapeutic benefits. Achieving these milestones could bridge the gap between experimental therapies and routine clinical use, transforming the management of ischemic heart disease.
